# Fitness cost and benefit of antimicrobial resistance in *Neisseria gonorrhoeae*: Multidisciplinary approaches are needed

**DOI:** 10.1371/journal.pmed.1002423

**Published:** 2017-10-31

**Authors:** Magnus Unemo, Christian L. Althaus

**Affiliations:** 1 WHO Collaborating Centre for Gonorrhoea and other STIs, Department of Laboratory Medicine, Faculty of Medicine and Health, Örebro University, Örebro, Sweden; 2 Institute of Social and Preventive Medicine, University of Bern, Bern, Switzerland

## Abstract

In a Perspective on the research article by Didelot and colleagues, Magnus Unemo and Christian Althaus discuss the value of modelling studies to inform antimicrobial resistance management and the limitations of the current evidence base informing such models.

Antimicrobial resistance (AMR) in *Neisseria gonorrhoeae* is a major public health concern globally, compromising the treatment of gonorrhea and its severe sequelae [1–3]. In response to emerging resistance to extended-spectrum cephalosporins (ESCs; cefixime and ceftriaxone), many countries introduced dual antimicrobial therapy for gonorrhea (mainly ceftriaxone plus azithromycin). Subsequently, resistance to the previous first-line treatment, cefixime, has decreased in many countries, including England [4,5]. However, several imperatives remain: increased surveillance of AMR (ideally including treatment outcomes and prescribing), rapid point-of-care tests that can simultaneously detect gonococci and predict resistance to multiple antimicrobials, novel antimicrobials with new mechanisms of action, and, ideally, an effective gonococcal vaccine [1–5]. Furthermore, improved understanding of the emergence, evolution, spread, and fitness of AMR in gonococci, which could predict future AMR scenarios and ideally mitigate them and inform public health practices, is also crucial but has received less attention.

In a research study in the latest issue of *PLOS Medicine*, Xavier Didelot and colleagues [6] aimed to estimate the fitness cost and benefit of cefixime resistance in gonococci in men who have sex with men (MSM) in England, using mathematical modeling and Bayesian inference. They developed a stochastic compartmental model representing the natural history and transmission of cefixime-sensitive and cefixime-resistant gonococcal strains. The authors assumed that the resistant strain has a fitness cost that results in faster clearance from asymptomatic infection. The model was then fitted to data on the total number of diagnoses and incidence of cefixime-resistant gonorrhea from 2008 to 2014. Briefly, the investigators estimated that cefixime treatment of cefixime-resistant strains was unsuccessful in 83% (95% credible interval [CrI] 53%–99%) of cases, resulting in a potential fitness benefit of resistance. This benefit was counterbalanced by the fitness cost when 31% (95% CrI 26%–36%) of patients were treated with cefixime, and when >55% (95% CrI 44%–66%) of patients were treated with cefixime, the resistant strain had a net fitness advantage over the susceptible strain. The study concludes that a previously abandoned antimicrobial such as cefixime could be reintroduced for treatment of a minority of gonorrhea patients without raising resistance levels.

## Alternative hypotheses

Didelot and colleagues’ study [6] had important aims and represents a valuable addition to the sparse literature on gonorrhea modeling specifically related to AMR. A major limitation of the study, however, is that the authors have not used their model to test alternative hypotheses that could explain the decline in cefixime resistance levels. In particular, introduction of dual therapy with ceftriaxone plus azithromycin, along with routine test-of-cure, in England in 2011 [7] might have effectively started to replace the *N*. *gonorrhoeae* multiantigen sequence typing (NG-MAST) G1407 lineage that accounted for most cefixime resistance and most of the verified cefixime treatment failures in England and many other countries [8–11]. G1407 was especially prevalent among MSM [8–10], and pharyngeal gonorrhea, which is effectively eradicated by dual therapy, might have previously acted as a reservoir. This hypothesis is consistent with the significant decrease in G1407 prevalence from 2009–2010 to 2013, associated with a significant decline in cefixime resistance among MSM, in England and in Europe in general [10]. In addition, this decline in G1407 prevalence resulted in an increased susceptibility to ceftriaxone [5,8–10], which is affected by the same ESC resistance mechanisms. The G1407 lineage appears to also have a relatively high fitness because the first G1407 isolate with ESC resistance determinants was identified in Japan in 2003 [12], and it has subsequently spread globally, accounting for most ESC resistance and treatment failures [8–11]. Additionally, a significant fitness cost resulting in rapidly decreasing cefixime resistance levels when cefixime is excluded from the recommended treatment is not in accordance with data from several other countries. Japan excluded cefixime and all other oral ESCs from the recommended treatment guideline in 2006, and resistance to cefixime has remained at a high level [13,14]. Therefore, in addition to a possible fitness cost, the decrease in cefixime resistance has most likely been highly affected by clonal replacements of G1407—and other gonococcal lineages associated with cefixime resistance—by cefixime-susceptible lineages, which has been driven by the discontinuation of cefixime and use of effective dual therapy (particularly the ceftriaxone component of the dual therapy).

## Limitations of the current evidence base

The state-of-the-art framework that Didelot and colleagues [6] used to infer the transmission dynamics of gonorrhea nicely illustrates the importance and power of such modeling studies. However, the study also shows the weak evidence base for several key assumptions and model parameters, resulting in large credible intervals that describe the transmission of sensitive and resistant strains and other complex epidemiological processes. First, the authors assume that the fitness cost solely acts through a reduction in the duration of asymptomatic infections, but it could also be associated with lower transmissibility or a higher proportion of symptomatic infections. Investigating the effects of these alternative fitness costs on the selection and spread of gonococcal resistance is considerably more complex [15]. It is also important to note that gonococcal AMR mechanisms do not necessarily cause significantly lower fitness, which results in the persistence of AMR and multidrug-resistant strains even in the absence of obvious antimicrobial pressure through recommended gonorrhea treatment guidelines. The reasons for this likely include that gonococci are effective in developing compensatory mutations that restore the possible fitness cost of AMR determinants. Some AMR determinants (e.g., *mtrR* and *gyrA* mutations) appear to be able to even enhance the fitness of at least some gonococcal strains. Thus, many years after penicillin, tetracycline, and fluoroquinolones were no longer recommended as treatments for gonorrhea, resistant strains continue to represent a significant percentage of isolates globally [16].

Second, the study considers a homogenous population of MSM and does not take into account heterogeneities in sexual behavior or practices, as others have done [15,17]. For some antimicrobials with frequently induced resistance, such as ciprofloxacin, the rate at which resistant gonorrhea spreads might be more affected by the treatment rate rather than by different levels of sexual activity, but only in the absence of a fitness cost [15]. Furthermore, the calculation of the basic reproduction number (*R*_0_) for sensitive and resistant strains heavily depends on assumptions about the level of heterogeneity in sexual behavior and sexual mixing [18]. Therefore, Didelot and colleagues’ findings about the appropriate levels of treatment with cefixime [6], which are based on results derived from *R*_0_, need to be treated with caution.

Third, we are lacking sufficient knowledge regarding the natural course, relative prevalence, transmissibility, probability of developing symptoms, duration of symptoms, average time to detection/treatment, effects of cotreatment, and pathogenicity of gonorrhea by anatomical site. Consequently, these complexities cannot be appropriately considered in mathematical models to date. Instead, the rate of transmission and the likelihood of developing symptoms used in models are frequently an assumption of the average for any infection site, which is not an ideal situation when these parameters differ significantly depending on the site of infection and sexual practice.

Fourth, improved data about the frequency of gonorrhea treatment failures and type of antimicrobial prescriptions (treatment and cotreatment) internationally are essential. For instance, when cefixime monotherapy was recommended from 2005 to 2010 in England (and Europe), azithromycin 1 g was also frequently administered empirically to eradicate concomitant *Chlamydia trachomatis* infection [19], and azithromycin 1 g by itself cures most gonorrhea cases [20]. Thus, dual therapy was also frequently administered before the introduction of the dual therapy for gonorrhea, particularly to the high-risk group of MSM and especially when pharyngeal gonorrhea was not excluded. Additionally, MSM are in general more frequently screened/tested (because of high risk or partner notification) and subsequently treated, and also, test of cure for these patients is not uncommon. Many gonorrhea cases among MSM would consequently have been detected and appropriately treated (with dual therapy or only ceftriaxone before or after test of cure) despite being asymptomatic, but Didelot and colleagues [6] made the assumption that asymptomatic infections were not treated and only resolved spontaneously or because of antimicrobials taken for other indications. As a result, the high level (83%) of treatment failures for cefixime-resistant gonorrhea predicted by the model, which also used the United Kingdom Gonococcal Resistance to Antimicrobials Surveillance Programme (GRASP) cefixime resistance breakpoint [4] and not the higher clinical cefixime resistance breakpoint stated by the European Committee on Antimicrobial Susceptibility Testing (EUCAST; www.eucast.org), is overestimated.

Finally, we need an enhanced microbiological understanding regarding the genetic predisposition of strains to develop resistance and the risk factors that are associated with emergence and evolution of AMR (patient, gonococcal strains, and coinfecting/commensal bacteria) to be able to take into account both direct transmission and de novo emergence of AMR and how the proportion of these mechanisms differ depending on the population and antimicrobial investigated. Many of these factors were not included in Didelot and colleagues’ model [6], mostly because of the lack of appropriate evidence.

It also needs to be noted that the authors legitimately emphasize that some of the aspects regarding limitations of the current evidence base mentioned above would likely influence the dynamics of cefixime-sensitive and cefixime-resistant strains in a similar manner and would not significantly affect the study’s main findings.

There are still few gonorrhea mathematical modeling studies specifically related to AMR, so additional studies are encouraged, particularly using multidisciplinary approaches that take into account microbiological, genomic, evolutionary, clinical, immunological, and epidemiologic data. Future modeling studies will be of value to inform AMR emergence and spread, management of gonorrhea, and public health practices. However, strengthening the evidence base that informs many key model assumptions and parameters remains imperative. Finally, ceftriaxone (which is affected by the same resistance determinants as cefixime) is our last remaining option for monotherapy of gonorrhea. Stronger multidisciplinary evidence and widely implemented management guidelines are essential before we recommend the reintroduction of monotherapy with cefixime, which has previously, and might in the future, select for resistance to ceftriaxone.

## Abbreviations

AMR: antimicrobial resistance
CrI: credible interval
ESC: extended-spectrum cephalosporins
EUCAST: European Committee on Antimicrobial Susceptibility Testing
GRASP: Gonococcal Resistance to Antimicrobials Surveillance Programme
MSM: men who have sex with men
NG-MAST: *Neisseria gonorrhoeae* multiantigen sequence typing
